# Multi-minicore Disease

**DOI:** 10.1186/1750-1172-2-31

**Published:** 2007-07-13

**Authors:** Heinz Jungbluth

**Affiliations:** 1 Department of Paediatric Neurology, Evelina Children's Hospital, St. Thomas' Hospital, Lambeth Palace Road, London, SE1 7EH, UK

## Abstract

Multi-minicore Disease (MmD) is a recessively inherited neuromuscular disorder characterized by multiple cores on muscle biopsy and clinical features of a congenital myopathy. Prevalence is unknown. Marked clinical variability corresponds to genetic heterogeneity: the most instantly recognizable classic phenotype characterized by spinal rigidity, early scoliosis and respiratory impairment is due to recessive mutations in the selenoprotein N *(SEPN1) *gene, whereas recessive mutations in the skeletal muscle ryanodine receptor *(RYR1) *gene have been associated with a wider range of clinical features comprising external ophthalmoplegia, distal weakness and wasting or predominant hip girdle involvement resembling central core disease (CCD). In the latter forms, there may also be a histopathologic continuum with CCD due to dominant *RYR1 *mutations, reflecting the common genetic background. Pathogenetic mechanisms of *RYR1*-related MmD are currently not well understood, but likely to involve altered excitability and/or changes in calcium homeoestasis; calcium-binding motifs within the selenoprotein N protein also suggest a possible role in calcium handling. The diagnosis of MmD is based on the presence of suggestive clinical features and multiple cores on muscle biopsy; muscle MRI may aid genetic testing as patterns of selective muscle involvement are distinct depending on the genetic background. Mutational analysis of the *RYR1 *or the *SEPN1 *gene may provide genetic confirmation of the diagnosis. Management is mainly supportive and has to address the risk of marked respiratory impairment in *SEPN1*-related MmD and the possibility of malignant hyperthermia susceptibility in *RYR1*-related forms. In the majority of patients, weakness is static or only slowly progressive, with the degree of respiratory impairment being the most important prognostic factor.

## Disease name(s)

Multi-minicore Disease; Minicore myopathy; Multicore myopathy; Multiminicore myopathy; Minicore myopathy with external ophthalmoplegia; Multicore myopathy with external ophthalmoplegia; Multiminicore disease with external ophthalmoplegia

## Definition

Multi-minicore Disease (MmD) is an inherited neuromuscular disorder defined by a) multiple areas with reduced oxidative activity running along an only limited extent of the longitudinal axis of the muscle fibre ("minicores") and b) clinical features of a congenital myopathy.

The condition was originally reported in a family with two affected siblings and suggestive histopathological findings [1]; various different designations ("Minicore myopathy", "Multicore myopathy", "Multiminicore myopathy", "Minicore myopathy with external ophthalmoplegia", "Multicore myopathy with external ophthalmoplegia", "Multiminicore disease with external ophthalmoplegia") have been assigned to cases with similar histopathological features and reflect the wide variability of both core appearance on muscle biopsy and associated clinical findings.

## Epidemiology

Epidemiological data are only available for the congenital myopathies as a group but not for individual conditions. The incidence of all congenital myopathies is estimated at around 0.06/1,000 live births, or onetenth of all cases of neuromuscular disorders [2]. Regional studies in Northern Ireland [3] and Western Sweden [4] suggest a prevalence between 3.5 – 5.0/100,000 in a Paediatric population.

Forms of MmD due to recessive mutations in the selenoprotein N *(SEPN1) *gene are probably rarer than those due to mutations in the skeletal muscle ryanodine receptor *(RYR1) *gene (personal observation), as mutations in the latter gene appear to be the most common genetic cause of a wide variety of congenital myopathies including central core disease (CCD) [5], certain forms of centronuclear myopathy (CNM) [6] and specific subgroups of MmD.

## Clinical description

Presentation of MmD is usually in infancy or childhood with hypotonia or proximal weakness; prenatal onset with reduced fetal movements and polyhydramnios has also been recognized [7,8]. Few cases with onset in adult life have been reported in the premolecular area, occasionally associated with progressive muscle weakness and respiratory or cardiac failure [9-12].

Clinical features associated with minicores on muscle biopsy are markedly heterogeneous:

spinal rigidity, scoliosis and respiratory impairment are the hallmark of the most instantly recognizable, classic phenotype of MmD [7,8,13]; onset is usually early in life and feeding difficulties with failure to thrive may be a presenting feature. A high-pitched voice and myopathic facial features are common, occasionally associated with a high-arched or cleft palate. Axial muscle weakness, particularly affecting neck and trunk flexors, is prominent, and failure to acquire head control may be an early sign. Proximal muscle groups, particularly those of the shoulder girdle, are more affected than distal muscles. Muscle wasting mainly affects axial groups, the shoulder girdle and the inner thigh ("bracket-like appearance"). A progressive scoliosis, often associated with lateral trunk deviation, and respiratory impairment have typically evolved by the second decade. Respiratory impairment may lead to secondary cardiac failure [7,8,14-16] and is often grossly out of proportion to the overall degree of weakness.

**Figure 1:**
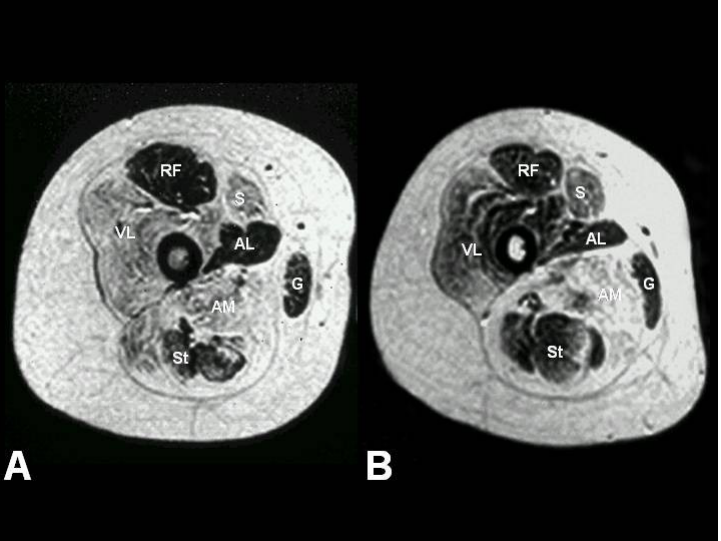
Selective muscle involvement in *RYR1*-related central core disease (CCD) and multi-minicore disease (MmD). Muscle MRI of the thigh, T1-weighted images. A) A transverse section from the proximal thigh in a 12-year-old patient with central core disease (CCD) due to a dominant mutation in the skeletal muscle ryanodine receptor *(RYR1) *gene. There is a distinct pattern of selective involvement characterized by marked increase in signal within the vasti, sartorius (S), and adductor magnus (AM), and relative sparing of the rectus femoris (RF), adductor longus (AL), gracilis (G), and hamstring muscles. B) A transverse section from the proximal thigh in a 17-year-old girl with Multi-minicore disease due to a homozygous recessive *RYR1 *mutation demonstrating a comparable pattern of selective involvement. * Reprinted from **[24] Neurology **2002 Jul 23;**59**(2):284–7. Jungbluth *et al*.: Autosomal-recessive inheritance of *RYR1 *mutations in a congenital myopathy with cores.**With permission from Lippincott Williams & Wilkins (LWW).**

A subset of patients with a distribution of weakness and wasting similar to the classic phenotype show additional extra-ocular muscle involvement (MmD with external ophthalmoplegia), pronounced on abduction and upward gaze, occasionally evolving over time [7,17-21]. However, respiratory impairment is usually milder than in the classic form, with the exception of the most severely affected neonatal cases [22]. Predominant hip girdle weakness with relative sparing of respiratory and bulbar muscles similar to the pattern in CCD is observed in another subgroup of MmD; some patients may show additional marked distal weakness and wasting, predominantly affecting the hands (moderate form of MmD with hand involvement) [23,24]. As in CCD, exercise-induced myalgia is common, and cryptorchism may be an additional feature in males (personal observation). The pattern of selective involvement on muscle imaging is similar to that observed in classic CCD caused by dominant *RYR1 *mutations [21,23-25] (Figure 1), and distinct from the selective muscle involvement described in myopathies due to recessive mutations in the *SEPN1 *gene [26]. The latter two groups may form part of a clinical spectrum rather than distinct entities, as suggested by the observation of extra-ocular muscle involvement evolving over time in patients with the moderate form of MmD [21]. Few severely affected cases have been reported with antenatal onset, generalized arthrogryposis, dysmorphic features and mild to moderate reduction of respiratory function [8].

Respiratory impairment is marked in the classic phenotype of MmD but usually mild or absent in other forms. Cardiac, mainly right ventricular impairment, is usually secondary to marked respiratory involvement [7] and therefore limited to the classic phenotype of MmD; congenital cardiac defects, particularly mitral valve prolapse, have been reported in few patients [1].

The association with malignant hyperthermia (MH) is not as well documented as in CCD due to dominant *RYR1 *mutations [27], but clinical MH episodes have been recognized in few cases with MmD [28,29]; minicores have also been noted in muscle biopsies from families with MH susceptibility due to *RYR1 *mutations but no other clinical features of a congenital myopathy [30,31]. Precautions during general anaesthesia such as avoidance of MH-triggering agents such as volatile anaesthetics or muscle relaxants should therefore be taken in MmD patients, particularly those with likely involvement of the *RYR1 *gene.

For diagnostic clues to aid the sometimes difficult distinction between *SEPN1*- and *RYR1*-related forms of MmD see Table 1.

**Table 1:** Clinical and histopathologic features which may aid the distinction between *SEPN1*-related and *RYR1*-related forms of Multi-minicore disease (MmD).

**Feature**	***SEPN1*-related MmD**	***RYR1*-related MmD**
**Clinical**		
Extraocular involvement	-	++
Bulbar involvement	+	+
Respiratory involvement	+++	+
Scoliosis	+++	++
Malignant hyperthermia susceptibility	-	+
		
**Histopathology**		
Type 1 predominance/uniformity	+	+++
Increase internal nuclei	+	+++
Multiple large cores ("multicores")	+	+++
Numerous small cores ("minicores")	+++	+

(-) = feature not reported, (+) = feature reported, (++) = common feature, (+++) = very common feature

## Aetiology

The marked clinical variability of MmD is reflected in genetic heterogeneity: Recessive mutations in both the selenoprotein N *(SEPN1) *[13] and the skeletal muscle ryanodine receptor *(RYR1) *gene [17,23-25,32] have been recently identified in clinically distinct subgroups; most reports of dominant inheritance predate molecular resolution of the condition [9,33-38] and may have been due to dominant mutations in the *RYR1 *gene or other genes giving rise to cores on muscle biopsy.

The selenoprotein N *(SEPN1) *gene on chromosome 1p36 was considered as a candidate for MmD because of the considerable clinical and histopathologic overlap between the classic phenotype of MmD (see paragraph above on clinical description) and congenital muscular dystrophy with rigidity of the spine (RSMD) due to *SEPN1 *mutations [39]; both conditions share a similar phenotype with marked axial weakness, spinal rigidity, early scoliosis and respiratory impairment, and histopathologic features of RSMD may be rather more myopathic than dystrophic, with typically a normal creatine kinase (CK). More than 30 *SEPN1 *mutations associated with a congenital myopathy phenotype have been identified to date and account for around 50% of cases with the classic phenotype of MmD [13]. *SEPN1 *mutations in RSMD and MmD are predominantly truncating, with few missense mutations typically affecting functionally important domains of the protein. Homozygous mutations are unexpectedly common even in families from non-consanguineous backgrounds, due to the presence of few founder mutations in different European populations. Selenoprotein N, a glycoprotein localized in the endoplasmic reticulum, belongs to a family of proteins mediating the effect of selenium [for review, [40]] and is involved in various antioxidant defence systems and several metabolic pathways. Abundant expression in fetal muscle precursor cells [41] indicates a role in myogenesis, also supported by the more recent finding of marked myofibrillar alterations in the zebrafish embryo following inhibition of the *SEPN1 *gene [42]. Although the precise function of selenoprotein N in muscle remains unclear, possible involvement in calcium homoeostasis is suggested by a structural motif similar to those found in calcium-binding proteins [39].

Clinical subgroups other than the classic phenotype of MmD have now been associated with recessive homozygous and compound heterozygous mutations in the skeletal muscle ryanodine receptor *(RYR1) *gene. Homozygous *RYR1 *mutations have been identified in consanguineous families of different ethnic background with the moderate form (with or without hand involvement) of MmD [23,24]; despite distinct histopathologic appearance, clinical features and findings on muscle imaging were similar to those observed in CCD secondary to heterozygous dominant *RYR1 *mutations. A cryptic splice site mutation in *RYR1 *intron 101 was identified in a severely affected isolated case from a consanguineous Tunisian family with MmD and ophthalmoplegia [22], resulting in a marked depletion of the normal *RYR1 *transcript, probably explaining the severe phenotype; both parents were asymptomatic carriers. *RYR1 *involvement was also suggested by linkage evidence and subsequent mutational analysis in four additional families with MmD and opthalmoplegia [17], including the original family reported by Swash and Schwartz [18]. In addition to compound heterozygosity for recessive *RYR1 *mutations in one case, heterozygous *RYR1 *mutations inherited from an asymptomatic father and expressed on a haploinsufficient background were identified in three families; this has now been attributed to epigenetic allele silencing of the *RYR1 *gene as a novel mechanism in the pathogenesis of core myopathies [43]. The *RYR1 *gene is also a likely candidate for the severe form of MmD with neonatal onset and arthrogryposis, considering phenotypic overlap with the form of CCD with fetal akinesia sequence [44].

**Figure 2:**
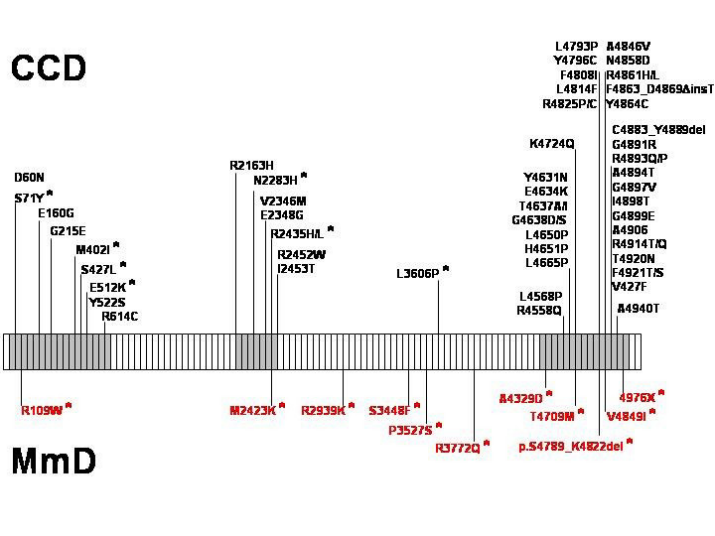
Schematic representation of the skeletal muscle ryanodine receptor *(RYR1) *gene and distribution of dominant and recessive (*) mutations associated with central core disease (CCD, in black) and Multi-minicore Disease (MmD, in red). Dominant mutations associated with a CCD phenotype predominantly affect the *RYR1 *C-terminal domain encoding the calcium release channel pore of the ryanodine receptor protein, whereas recessive mutations predominantly associated with a MmD phenotype are distributed evenly throughout the gene. N-terminal, central and C-terminal mutational hotspots within the *RYR1 *gene are highlighted in grey. (Figure courtesy of Dr Haiyan Zhou)

*RYR1 *is organized in 106 exons [45] and encodes the skeletal muscle ryanodine receptor (RyR1), the principal, ligand-gated sarcoplasmic reticulum calium release channel with a crucial role in excitation-contraction (E-C) coupling by regulating cytosolic Ca^2+ ^levels. RyR1 calcium release is primarily triggered by voltage-induced conformational changes of the abutting dihydropyridine (DHPR) receptor, and secondarily by a number of exogeneous and endogeneous effector molecules [for review, [46]]. RyR1 N-terminal portions are myoplasmic and constitute the visible foot structure that interacts with the DHPR receptor, whereas the actual calcium release channel is located in the C-terminal part of the protein [47]. The large majority of the more than 100 *RYR1 *mutations identified to date were dominant mutations associated with malignant hyperthermia susceptibility (MHS) and central core disease (CCD) phenotypes, typically clustered in the cytoplasmic N-terminal (MHS/CCD region 1, amino acids 35 – 614; mainly MHS), the central (MHS/CCD region 2, amino acids 2163 – 2458; mainly MHS) and the C-terminal (MHS/CCD region 3, amino acids 4550 – 4940; mainly CCD) domains of the protein [for review, [48]; also [32,49]]. In contrast, recessive *RYR1 *mutations associated with MmD have so far only been identified in a small number of families [17,22-24,32] and appear to be distributed throughout the entire *RYR1 *gene (Figure 2); compound heterozygosity for mutations affecting MHS-related domains of the protein associated with a MmD phenotype has been recognized [17,32]. The majority of MmD-related *RYR1 *mutations were missense mutations; few intronic splicing mutations have been recently reported [17,22,32].

In contrast to *RYR1 *mutations associated with CCD and MHS, MmD-associated *RYR1 *mutations have only been recently studied and specific functional consequences are currently only imperfectly understood. The functional effects of *RYR1 *mutations associated with CCD and MHS have been extensively investigated in response to the *in vitro *contracture test (IVCT), in cultured myotubes from patients and in various homologous and heterologous expression systems, resulting in a tentative understanding of the molecular pathophysiology implicated in those phenotypes [for review, [48]]. MH-related *RYR1 *mutations are thought to result in hypersensitivity of the RyR1 channel, lowering the activation threshold to lower agonist concentrations, whereas CCD-related *RYR1 *mutations appear to reflect defective intracellular Ca^2+ ^homoeostasis, either due to depletion of intracellular Ca^2+ ^stores ("leaky channel hypothesis") [50] or because of impaired channel efficiency at transporting Ca^2+ ^after activation *via *depolarization ("E-C uncoupling hypothesis") [51].

The few functional data available to date on MmD-related *RYR1 *mutations [52-54] suggest a wide variety of pathogenetic mechanisms underlying this phenotype: based on studies of Ca^2+ ^homoeostasis in EBV-immortalized lymphoblasts from patients [52], the previously reported homozygous P3527S substitution [23] was demonstrated to result in decreased stimulated Ca^2+ ^release despite preserved intracellular Ca^2+ ^stores (suggestive of an uncoupled channel), whereas the V4849I substitution [24] was associated with a small but significant effect on resting Ca^2+ ^concentrations. E-C uncoupling was also indicated by complete loss of Ca^2+ ^conductance in recombinant mutant RyR1 channels expressing a Arg109Trp substitution [53] previously associated with a MmD and ophthalmoplegia phenotype [17]. Marked reduction of the amount of the RyR1 protein in some individuals [22,32] suggests that MmD-related *RYR1 *mutations may affect protein expression and stability as much as function. Additional work will be required to further elucidate the molecular mechanisms underlying *RYR1*-related MmD, and may contribute to the understanding of E-C coupling and RyR1 interactions.

## Diagnostic methods

Multi-minicore Disease (MmD) is a histologic diagnosis established on **muscle biopsy**. MmD is characterized by multifocal, well-circumscribed areas with reduction of oxidative staining and low myofibrillar ATPase [1].

**Figure 3:**
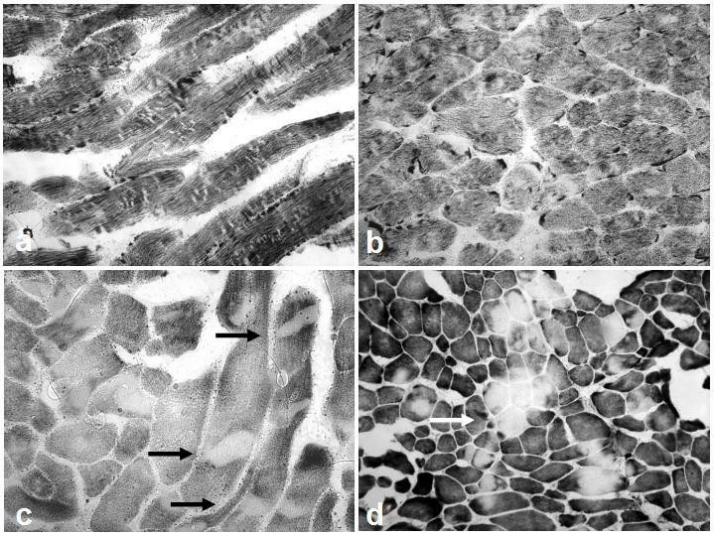
Histopathological features of Multi-minicore disease. NADH-TR (a–c) and cytochrome oxidase (COX) (d) stains, horizontal (a,c) and transverse (b,d) sections from a 3-year-old (a–b) and a 9-year-old girl (c–d) from different families. Predominance of darker staining type 1 fibres is prominent in both patients, whilst appearance of core lesions is widely variable, ranging from numerous small lesions of limited extent ("minicores") (a-b) to few multiple large lesions often extending throughout the entire fibre diameter ("multicores") (c, →) and occasionally affecting the same area in adjacent fibres (d, →). Whilst "minicores" are more frequently found in *SEPN1*-related MmD, the latter appearance is more typical of the *RYR1*-related form and indicates a possible histopathologic continuum with central core disease (CCD) due to mutations in the same gene.

In contrast to central cores, minicores extend only for a short distance along the longitudinal axis of the muscle fibre, are typically unstructured and may affect both type 1 and type 2 fibres [55]; a peculiar picture described as "focal loss of crossstriations" in muscle fibres has been reported in some families [18,19]. Cores may vary substantially in size and morphology (Figure 3), to some extent depending on the genetic background: *SEPN1*-related MmD is typically associated with numerous small lesions scattered throughout the muscle fibre ("minicores"), whereas multiple larger lesions ("multicores") are more commonly seen in forms of MmD related to recessive mutations in the *RYR1 *gene; in the latter group, there may be a continuum with the histopathologic appearance of CCD, occasionally evolving over time as demonstrated in patients on consecutive muscle biopsies [23,56]. Predominance or uniformity of hypotrophic type 1 fibres are commonly associated and may precede the appearance of more specific features [7,56,57]; these findings appear to be more commonly observed in cases due to *RYR1 *mutations compared to *SEPN1*-related MmD, where fibre typing is often preserved (Sewry, personal observation). A potential overlap with the milder end of the congenital muscular dystrophy spectrum suggested by the observation of whorled fibers, an increase in fat and connective tissue and more dystrophic changes in some individuals [13,55] was recently genetically confirmed by the identification of recessive *SEPN1 *mutations in cases of classical MmD and muscular dystrophy with early rigidity of the spine, a mild form of congenital muscular dystrophy [13,39].

On electron microscopy, minicores present as areas of myofibrillar disruption and paucity of mitochondria, often with degeneration of the sarcomeres and structural changes of the sarcoplasmic reticulum and transverse tubules [55]. The same biopsy may show different stages of minicore formation, ranging from Z-line streaming with preserved myofibrillar structure to areas with complete loss of the sarcomeric architecture [1].

Limited immunohistochemical studies in MmD suggest sarcoplasmic reticulum and desmin abnormalities corresponding to those observed in CCD [58]; as with central cores, minicores stain also strongly positive with antibodies to the actin cross-linking protein filamin C in a non-specific manner [59]. A more recent study suggests disturbance of Ca^2+ ^-related proteins as a useful immunohistochemical marker to distinguish *RYR1*-related forms from *SEPN1*-related forms of MmD [60].

In cases with equivocal clinical or histopathological features, **muscle MR imaging **may complement clinical assessment and aid the choice of appropriate genetic testing, as the pattern of selective muscle involvement associated with *RYR1 *mutations [25] (Figure 1) is distinct from that observed in *SEPN1*-related cases [26] or other congenital myopathies such as nemaline myopathy [61].

## Differential diagnosis

The finding of cores on muscle biopsy is non-specific and may be observed in a number of different contexts, including clinical conditions with no or little associated weakness and as an additional finding in other neuromuscular disorders.

**Minicores as a non-specific finding on muscle biopsy **have been reported in healthy probands following eccentric exercise (*i.e*., lengthening during activity) [62] and on muscle biopsies from families with MH susceptibility due to *RYR1 *mutations but no other clinical features of a congenital myopathy [30,31]; they may occur in other contexts such as dystrophy, denervation, inflammatory and endocrine myopathies [for review, [1]] and primarily metabolic conditions such as type III glycogenosis [63] or short-chain acyl-CoA dehydrogenase (SCAD) deficiency [64]. It is therefore important to emphasize that the presence of minicores on muscle biopsy without associated weakness or in the presence of other disease causes is not sufficient to constitute a diagnosis of MmD. Single MmD families have been reported with multiple pterygium syndrome [65] and a complex dysmorphic syndrome featuring short stature, musculoskeletal anomalies, mental retardation, and pituitary hypoplasia with hypogonadotrophic hypogonadism [66]; the genetic basis for this association is currently unclear.

The occurrence of minicores on muscle biopsy in association with features of **other congenital myopathies **such as central cores, nemaline rods or central nuclei has been well-documented in the pre-molecular era [14,33,67-72] and is currently only partially genetically resolved. Core-like structures, with or without additional nemaline rods, have been reported in association with dominant mutations in the *ACTA1 *gene [73,74], and there is recent evidence that recessive mutations in the *RYR1 *gene may give rise to the appearance of centronuclear myopathy with or without additional cores on muscle biopsy [6]. As minicores are thus not specific, the diagnosis of MmD requires the presence of minicores as the predominant feature on muscle biopsy in conjunction with suggestive clinical features.

The distinction from **central core disease (CCD) **due to dominant *RYR1 *mutations may be particularly challenging in the subset of MmD due to recessive mutations in the same gene, as histopathologic features in the latter groups and CCD may represent part of a continuous histopathologic spectrum rather than distinct entities [56]. However, MmD-associated recessive *RYR1 *mutations give rise to clinical features such as external ophthalmoplegia, bulbar involvement and a moderate degree of respiratory impairment not commonly observed in typical dominant CCD. Although recessively inherited *RYR1 *mutations appear to be more closely associated with the histopathologic appearance of MmD rather than CCD, dominant *RYR1 *mutations may occasionally give rise to minicores on muscle biopsy [56] and may have accounted for the small number of MmD pedigrees with apparent autosomal dominant inheritance reported in the pre-molecular area. In addition, the histopathologic appearance of *RYR1*-related MmD may evolve into the classic picture of CCD on follow-up biopsies later in life [23].

Although cardiac impairment secondary to respiratory involvement is not uncommonly observed in *SEPN1*-related MmD, primary cardiomyopathies have not been reported in genetically confirmed cases of MmD due to mutations in the *SEPN1 *or *RYR1 *genes. However, some **myopathies with primary cardiac involvement **and a distinct genetic background may feature minicores as an additional histopathologic finding. A rare autosomal dominant myopathy with associated dilated cardiomyopathy and both central and minicores on muscle biopsy has been attributed to mutations in the skeletal muscle **α**-actin *(ACTA1) *gene [74], whereas autosomal recessive mutations in the titin *(TTN) *gene were recently identified in a novel early-onset myopathy with minicores, increased central nuclei, dystrophic features and a fatal cardiomyopathy [75].

In addition, prominent desmin accumulation in skeletal and cardiac muscle fibres from other patients with minicores on muscle biopsy and a primary cardiomyopathy [76] may point to primary involvement of the desmin *(DES) *or related genes; also, mutations in the lamin A/C *(LMNA) *gene, a common cause of various muscular dystrophy and cardiac phenotypes [for review, [77]], may also be associated with core-like structures on muscle biopsy and have to be considered in cases with minicores and prominent cardiac involvement. It is likely that the few families with primary cardiomyopathies in association with minicores on muscle biopsy reported in the pre-molecular area [9,11,34,76,78] may have carried mutations in above genes rather than the *SEPN1 *and *RYR1 *genes now firmly associated with MmD.

## Management

No curative treatment is currently available for MmD and management is mainly supportive based on a multidisciplinary approach.

Regular physiotherapy is aimed at promoting endurance, preservation of muscle function and the prevention of contractures, particularly those of the tendon Achilles. Marked axial involvement is common, particularly in the classic form of MmD secondary to *SEPN1 *mutations, and exercises promoting truncal stability such as swimming and riding [79] may be particularly recommended. Scoliosis is almost invariable in the classic form of MmD and in most cases eventually will have to be managed surgically, considering that conservative approaches are often unsuccessful due to the progressive nature of the deformity. As in other neuromuscular conditions, mobilization following surgery ought to be early in order to avoid the detrimental effects of prolonged immobilization on muscle strength. Independent ambulation may be promoted by appropriate rehabilitative measures such as provision of weight bearing calipers in the most severe cases where walking can not be achieved without additional support.

Severe respiratory impairment is almost invariable in the classic phenotype of MmD secondary to *SEPN1 *mutations and has to be anticipated with a high degree of suspicion, as most patients remain independently mobile with only little functional impairment whilst already developing respiratory failure [7,8]. Patients have to be specifically questioned for symptoms suspicious of nocturnal hypoventilation such as early morning headaches, loss of appetite and daytime drowsiness; respiratory capacity ought to be monitored regularly and annual overnight oxygen saturation studies performed if forced vital capacity (FVC) is less than 60% of the expected value, and more frequently if FVC is less than 40% [80]. Respiratory impairment in other forms of MmD due to *RYR1 *mutations is usually less severe or absent, but considering the not negligible risk monitoring of respiratory function according to the principles outlined above is advisable. In all forms of MmD, respiratory infections should be treated actively.

Right ventricular impairment secondary to respiratory failure has been reported in the classic phenotype of MmD and patients with those clinical features ought to have regular cardiac assessments including cardiac ultrasounds. A primary cardiomyopathy has not yet been reported in patients with confirmed mutations in the *SEPN *or *RYR1 *genes; however, cores on muscle biopsy are non-specific and may be found as an additional feature in patients with primary cardiomyopathies due to mutations in the *DES*, *TTN*, *LMNA *and *ACTA1 *genes (see paragraph on differential diagnosis). Cardiac ultrasound studies therefore ought to be considered in cases where clinical features are unusual and mutations in the *SEPN1 *and *RYR1 *genes have been excluded.

Patients with MmD secondary to mutations in the *RYR1 *gene may be at risk of malignant hyperthermia reactions, an abnormal response to muscle relaxants such as succinylcholine and volatile anaesthetics [27,81], as has been reported in few cases [28,29]; minicores on muscle biopsy have also been noticed in few patients with *RYR1*-related MH susceptibility but no other clinical features of a congenital myopathy [30,31]. Although the association with MH susceptibility is not as clearly documented as in CCD and has been excluded by *in vitro *contracture testing in some families [23], precautions such as avoidance of potentially MH-triggering agents and availability of the RyR1 antagonist dantrolene should be taken during general anaesthesia.

The **β**-agonist salbutamol has been recently studied as a pharmacological agent in the treatment of *RYR1*-related myopathies with encouraging results [82], however, results of this pilot study will have to be validated in a larger randomized controlled trial as a basis for future recommendations.

## Genetic counselling

Genetic counselling should be offered to all families and individuals in whom a diagnosis of MmD has been made. *SEPN1*-related MmD and the majority of cases associated with mutations in the *RYR1 *gene are inherited as an autosomal recessive trait; only few families with multi-minicores on muscle biopsy and dominant inheritance have been documented.

Mutational analysis of the *SEPN1 *gene is now offered as a diagnostic service in most countries. Diagnostic *RYR1 *screening of selected exons has been established for malignant hyperthermia patients by a number of laboratories associated with the European Malignant Hyperthermia Group (EMHG) [83] however, patients with MmD are likely to require sequencing of the entire *RYR1 *coding sequence currently not widely available. Unlike mutations associated with a CCD phenotype, MmD-related *RYR1 *mutations appear to be distributed throughout the *RYR1 *coding sequence [17,32] and a mutational strategy focusing on mutational hotspots is therefore not feasible. The recent finding of haploinsufficiency secondary to epigenetic allele silencing in *RYR1*-related phenotypes [43] poses a particular challenge and should prompt screening of muscle-derived cDNA in cases where an affected child has inherited a mutation from an unaffected parent.

## Prognosis

Respiratory impairment is the main prognostic factor in *SEPN1*-related MmD and has to be managed pro-actively; patients can usually be maintained on nocturnal non-invasive ventilation for many years without major functional deterioration.

The forms of MmD due to recessive *RYR1 *mutations are usually associated with a mild to moderate degree of disability and carry an overall favourable prognosis, and, with the exception of the most severely affected neonatal cases, almost all patients achieve the ability to walk independently. The course of *RYR1*-related MmD in childhood and adolescence is static or only slowly progressive [17,32], but the absence of larger long term follow-up series makes it difficult to comment on the further course into adulthood.

## Unresolved questions

The precise molecular mechanisms underlying MmD are currently only partially understood. Recent limited functional studies suggest that recessive *RYR1 *mutations may affect function of the tetrameric RyR1 protein only in the homozygous state; this may be modulated through the effect on the binding of accessory and regulatory proteins involved in channel kinetics. More extensive functional studies are required to further delineate specific genotype-phenotype correlations in MmD and are likely to advance our understanding of excitation-contraction (E-C) coupling as a potential basis for future rational therapeutic approaches.

While the functional effects of *RYR1 *mutations associated with CCD, MHS and MmD are at least partially understood, the precise molecular mechanisms in the pathogenesis of *SEPN1*-related MmD remains unclear; a calcium-binding motif within the selenoprotein N protein suggests that intracellular Ca^2+ ^handling may also be affected in this subgroup of MmD and points at a common pathway in the pathogenesis of core formation.

A proportion of patients with typical features of MmD do not harbour mutations in the *RYR1 *or *SEPN1 *genes and this group is still awaiting genetic resolution.
